# Differential effects of energy stress on AMPK phosphorylation and apoptosis in experimental brain tumor and normal brain

**DOI:** 10.1186/1476-4598-7-37

**Published:** 2008-05-12

**Authors:** Purna Mukherjee, Tiernan J Mulrooney, Jeremy Marsh, Derek Blair, Thomas C Chiles, Thomas N Seyfried

**Affiliations:** 1 Department of Biology, Boston College, Chestnut Hill, Massachusetts 02467, USA

## Abstract

**Background:**

AMP-activated protein kinase (AMPK) is a known physiological cellular energy sensor and becomes phosphorylated at Thr-172 in response to changes in cellular ATP levels. Activated AMPK acts as either an inducer or suppressor of apoptosis depending on the severity of energy stress and the presence or absence of certain functional tumor suppressor genes.

**Results:**

Here we show that energy stress differentially affects AMPK phosphorylation and cell-death in brain tumor tissue and in tissue from contra-lateral normal brain. We compared TSC2 deficient CT-2A mouse astrocytoma cells with syngeneic normal astrocytes that were grown under identical condition *in vitro*. Energy stress induced by glucose withdrawal or addition of 2-deoxyglucose caused more ATP depletion, AMPK phosphorylation and apoptosis in CT-2A cells than in the normal astrocytes. Under normal energy conditions pharmacological stimulation of AMPK caused apoptosis in CT-2A cells but not in astrocytes. TSC2 siRNA treated astrocytes are hypersensitive to apoptosis induced by energy stress compared to control cells. AMPK phosphorylation and apoptosis were also greater in the CT-2A tumor tissue than in the normal brain tissue following implementation of dietary energy restriction. Inefficient mTOR and TSC2 signaling, downstream of AMPK, is responsible for CT-2A cell-death, while functional LKB1 may protect normal brain cells under energy stress.

**Conclusion:**

Together these data demonstrates that AMPK phosphorylation induces apoptosis in mouse astrocytoma but may protect normal brain cells from apoptosis under similar energy stress condition. Therefore, using activator of AMPK along with glycolysis inhibitor could be a potential therapeutic approach for TSC2 deficient human malignant astrocytoma.

## Background

AMP-activated protein kinase (AMPK) is a primary regulator of the cellular response to lowered ATP levels in eukaryotic cells [1,2]. AMPK is a serine/threonine protein kinase and a member of the Snf1/AMPK protein kinase family [1]. The activity of AMPK requires phosphorylation of the alpha subunit on Thr-172 in its activation loop by one or more upstream kinases (AMPKK) [3-5]. AMPK phosphorylation down regulates ATP consuming processes like the synthesis of fatty acids, cholesterol, and proteins, while up-regulating ATP producing catabolic pathways like fatty acid oxidation and glucose uptake. Previous studies indicate that AMPK has both pro and anti-apoptotic effects on eukaryotic cells [6-16]. The pro-apoptotic action of activated AMPK is associated with activation of stress kinases and caspase-3 [7-9,13,17]. In primary b cells, prolonged stimulation of AMPK induces apoptosis through an activation of c-Jun-N-terminal kinase (JNK) [13]. On the other hand, pharmacological activation of AMPK protects thymocytes from dexamethasone-induced apoptosis [11] and Rat-1 fibroblasts from serum withdrawal induced apoptosis [12].

AMPK is an anti-growth molecule because of its relationship with two tumor suppressor genes: LKB and TSC2 (tuberous sclerosis complex 2). LKB mutations cause Peutz-Jeghers syndrome, an autosomal dominant disorder characterized by multiple hamartomatous polyps in colon and other parts of the gastrointestinal tract [18-20]. LKB1 is the upstream activating kinase for the stress-responsive AMP-activated kinase, and provides a link between regulators of cellular metabolism and cell proliferation in cancer [21]. There are several reports that LKB1 activates AMPK and thus serves as the principal AMPKK [6,22,23]. LKB1 protects cells from apoptosis in response to agents that elevate intracellular AMP. LKB1-deficient mouse embryonic fibroblasts are defective in AMPK activation and undergo apoptosis under conditions that elevate AMP. Recent observations indicate that Ca2+/calmodulin – dependent protein kinase kinases also regulate AMPK in cell lines lacking expression of LKB1 [24,25].

Under energy stress, AMPK phosphorylates TSC2 on T-1227 and S-1345 which regulate cell size [26]. Activation of TSC2 by AMPK- dependent phosphorylation prepares cells for an unfavorable growth environment and protects cells from death. Glucose deprivation induces apoptosis in TSC2-/- and TSC1-/- cells [26]. Since mTOR is a key downstream target of TSC1/TSC2, inhibition of mTOR by rapamycin suppresses apoptosis in those cells following energy depletion [26]. LKB1 may activate TSC2 via the AMPK [26,27]. mTOR inhibitors have anti-neoplastic potential since mutations in LKB1, TSC2, or PTEN tumor suppressor genes produce aberrant mTOR activation in certain neoplastic conditions [28-31].

Dagon et al [16] recently found that a 40% dietary restriction increased hippocampal AMPK activity, induced neurogenesis, and improved cognition. On the other hand, a 60% dietary restriction over activated AMPK, reduced cognition, and induced neural apoptosis. We show here that phosphorylation of AMPK at Thr-172 produced apoptosis in TSC2 deficient CT-2A mouse astrocytoma under 40% dietary caloric restriction, whereas less AMPK phosphorylation with no apoptosis was seen in contra-lateral normal brain. This finding was further supported by *in vitro *observations where energy stress produced differential phosphorylation of AMPK in CT-2A cells and in astrocytes and where phosphorylation of AMPK enhanced apoptosis in CT-2A cells.

## Results

### Differential effects of energy stress on ATP depletion, AMPK activation and apoptosis in CT-2A tumor cells and normal astrocytes *in vitro*

**Figure 1:**
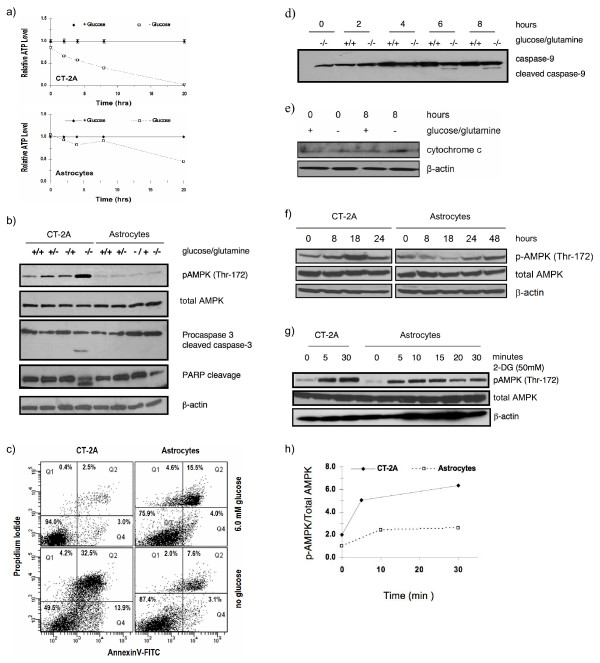
**Effects of energy stress on ATP depletion, AMPK phosphorylation and apoptosis in CT-2A and astrocyte cell lines**. a) Influence of glucose and/or glutamine deprivation on ATP levels in CT-2A astrocytoma and mouse astrocytes. Cells were grown in medium with glucose and glutamine. At 0 hour the medium was replaced with fresh medium without glucose and glutamine and ATP levels were measured at several time points between 0 and 20 hours. ATP levels are presented as relative level normalized to ATP levels in control cells that were given fresh medium with glucose and glutamine at 0 hour. b) Influence of glucose and/or glutamine deprivation on AMPK phosphorylation and caspase-3 cleavage in CT-2A and astrocytes. Cells were treated with different media as indicated for 24 hours. Cell lysates were probed with specific phosphorylated and non-phosphorylated antibodies for western blot as indicated. Increased phosphorylation of AMPK in CT-2A cell was evident with caspase-3 and PARP cleavage in complete absence of serum, glucose, and glutamine. c) Influence of glucose and/or glutamine deprivation on Annexin positive apoptotic cells. Cells were incubated in the presence and absence of glucose and glutamine containing media for 18 hours. Cells positive either for Annexin V or for both Annexin V and PI were considered early apoptotic and late apoptotic cells, respectively. Glucose/glutamine withdrawal induced apoptosis in 48–50% of CT-2A cells but not in astrocytes. d) Influence of glucose and/or glutamine deprivation on caspase 9 cleavage in CT-2A cells. Cells were treated with different media as indicated and incubated for 0–8 hours. Cell lysates were probed with anti-caspase 9 for western blot. Cleavage of caspase-9 started at 6 hours in complete absence of glucose and/or glutamine. e) Caspase-9 cleavage was associated with cytochrome c release at 8 hours in the cytosolic fraction of CT-2A cells. f) Influence of 3.0 mM glucose on AMPK phosphorylation in CT-2A and astrocytes. Cells were treated for different hours as indicated. AMPK was maximum phosphorylated in CT-2A cells at 18 hours and started dying in 24 hours. AMPK phosphorylation was greater in CT-2A than in astrocytes. g) Influence of 2-deoxyglucose (2-DG) on AMPK phosphorylation in CT-2A and astrocytes. Cells were treated with 50 mM conc. of 2-DG from 0–30 minutes in serum free media. Western blot analysis for phosphorylated AMPK revealed increased phosphorylation in CT-2A cells compared to astrocytes. h) Increase in AMPK phosphorylation with time (analyzed from g). All experiments were done in triplicate.

AMPK is activated by cellular ATP depletion in response to energy stress. To study energy stress induced ATP depletion and AMPK phosphorylation in the CT-2A tumor cells and in the control B6 mouse astrocytes, we removed either glucose and/or glutamine from the media for 24 hours. Since glutamine is the most abundant carbon compound in culture media next to the glucose, we removed both glucose and glutamine to ensure maximum energy stress on cells. We found that ATP depletion and AMPK phosphorylation at Thr-172 was significantly greater in the CT-2A cells than in the astrocytes under glucose or glutamine deprivation (Figure 1a, b). This energy stress also enhanced cleavage of caspase-3 and PARP in CT-2A cells within 24 hours indicating enhanced apoptosis (Figure 1b). On the other hand, low AMPK phosphorylation at Thr-172 was observed in astrocytes after 24 hours under similar energy stress (Figure 1b).

To determine the number of apoptotic cells, we stained the cells with Annexin-V, which specifically labels apoptotic cells. Figure 1c shows that glucose withdrawal induced apoptosis in 48% of CT-2A cells at 18 hours. A significant number of astrocytes were found Annexin and PI positive in the glucose containing medium which could be due to their high basal turnover rate and confluency. Glucose withdrawal had no significant effect on apoptosis in astrocytes. The increased number of living astrocytes was likely due to decrease in their proliferation rate in glucose free media. Caspase-3 activation in CT-2A cells was preceded by upstream caspase-9 cleavage after 6 to 8 hours of glucose and glutamine deprived conditions (Figure 1d). Caspase-9 cleavage was associated with cytochrome c release in the cytosolic part of CT-2A cells in 8 hours of glucose/glutamine free conditions (figure 1e). Caspase-9 cleavage and cytochrome c was not detected in astrocytes in the indicated time period (data not shown). Treatment of CT-2A cells with 3.0 mM glucose caused a marked elevation of AMPK phosphorylation in 18 hours, whereas astrocyte was stressed in low glucose condition in 48 hours (Figure 1f). CT-2A cells usually die after 48 hours with 3.0 mM glucose. Treatment of astrocytes with 3.0 mM glucose caused no AMPK phosphorylation at Thr-172 in 18 hours. AMPK phosphorylation was observed in both CT-2A and astrocytes following treatment with 2-DG, which inhibits glucose utilization (Figure 1g and 1h). In CT-2A, phosphorylation was significantly greater than in astrocytes following 2-DG treatment. These results indicate that reduced energy substrates produced significantly greater stress and apoptosis in CT-2A tumor cells than in normal astrocytes.

### AMPK stimulation caused apoptosis in CT-2A cells

**Figure 2:**
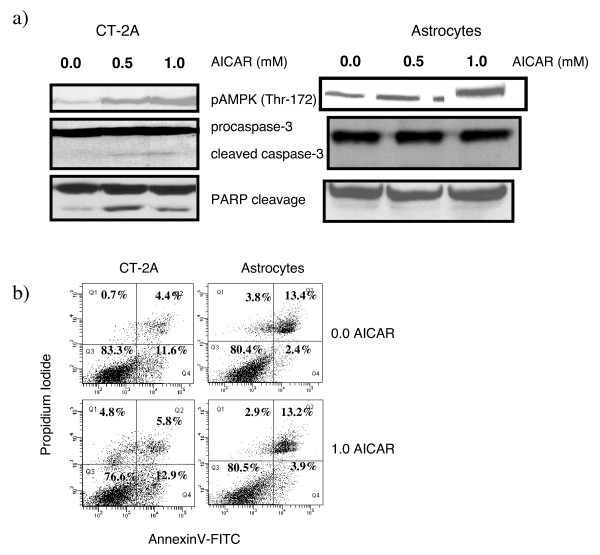
**Influence of AICAR on AMPK phosphorylation and apoptosis in CT-2A cells**. CT-2A and astrocyte cells were treated with AICAR (0.5 and 1.0 mM) in glucose, glutamine and serum containing media for 24 hours. a) Cell lysates were probed with specific phosphorylated and non-phosphorylated antibodies for western blot as indicated. Dose dependent increase in AMPK phosphorylation was evident in CT-2A and astrocytes but caspase-3 and PARP cleavage was noted only in AICAR treated CT-2A cells. b) Cells were treated with 1.0 mM AICAR in glucose, glutamine and serum containing media for 24 hours and treated with Annexin V and PI stain as described in methods. Labeled cells were analyzed by flow cytometry. 8–10% apoptotic cells were noted in CT-2A cells treated with AICAR compared to untreated cells. No significant cell-death was noticed in treated astrocytes. All experiments were done in triplicate.

To determine the effects of pharmacological stimulation of AMPK on CT-2A and astrocytes, cells were incubated with 0.5 and 1.0 mM AICAR in glucose and serum containing medium for 24 hours. AICAR is a cell permeable drug that is converted to AICAR monophosphate (ZMP) intracellularly and that mimics the stimulatory effect of AMP on AMPK. A dose dependent increase in AMPK phosphorylation at Thr-172 was observed in CT-2A and astrocytes (Figure 2a) whereas caspase-3 and PARP cleavage was observed only in CT-2A cells under normal energy conditions (figure 2a). Since AICAR was added in normal growth media, only 8–10% increase in Annexin positive apoptotic CT-2A cells were observed (Figure 2b). Taken together, these findings indicate that AMPK phosphorylation in CT-2A cells is associated with apoptosis.

### Differential effects of energy stress on AMPK activation and apoptosis in CT-2A brain tumor and normal brain *in vivo*

**Figure 3:**
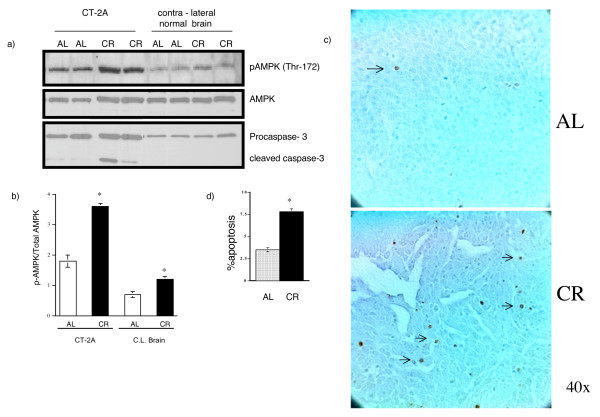
**Effects of energy stress on AMPK phosphorylation and apoptosis in CT-2A tumor and normal brain**. a) Influence of 40% dietary caloric restriction on AMPK phosphorylation and caspase-3 cleavage in CT-2A tumor grown orthotopically in B6 mice and in contra-lateral normal brain tissue. Tumor implantation, diet administration and collection of tumor and brain tissues were described in methods. Tissue lysates were analyzed with indicated antibodies by western blot. Results indicate increased AMPK phosphorylation and caspase-3 cleavage in the tumors of CR mice than in AL mice. Normal brain had much less AMPK phosphorylation compared to CT-2A tumor. Tissues from 3 independent mice for each group were analyzed. b) Ratio of AMPK phosphorylation to total phosphorylation. Asterisk indicates a significant increase in AMPK phosphorylation at p < 0.01. c) Influence of 40% dietary caloric restriction on apoptotic cells in CT-2A tumor grown orthotopically. Tumor implantation and diet treatment was the same as mentioned above. After 11 day, brain was dissected out and fixed in formalin. Paraffin embedded tissue was processed for TUNEL staining. Results indicate that the TUNEL positive apoptotic cells (black arrows) are higher in CR tumor than AL tumor. Surrounding normal brain has no significant cell-death. d) Tissues from 3 independent mice for each group were analyzed. Apoptotic cell index was significantly higher in CR tumor at p < 0.0001.

A 40% dietary caloric restriction (CR) was initiated in B6 mice 48 hours after orthotopic implantation of the CT-2A tumor in order to determine the effects of energy stress on AMPK phosphorylation in tumor and contra-lateral normal appearing brain tissue. Similar to *in vitro *findings, AMPK phosphorylation was significantly greater in the CT-2A tumor than in the normal brain tissue and CR caused a marked elevation of AMPK phosphorylation in CT-2A tumor and in normal brain (Figure 3a and 3b). AMPK phosphorylation was associated with caspase-3 cleavage in tumors, but not in normal brain (Figure 3a). A significant increase in TUNEL positive cells was found in CR tumors compared to AL tumors (Figure 3c and 3d). CR did not induce apoptosis in normal brain tissue. These *in vivo *findings support the *in vitro *findings and suggest that moderate CR is pro-apoptotic to brain tumors, but not to normal brain.

### Effects of energy stress on the mTOR/S6K protein synthesis pathway in CT-2A tumor cells and astrocytes

**Figure 4:**
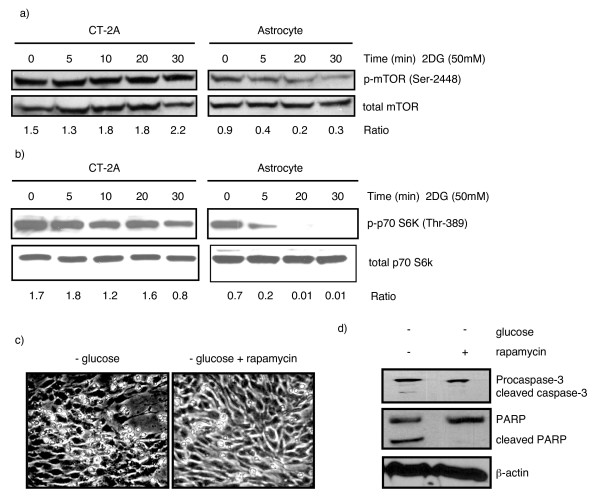
**Effects of energy stress on protein synthesis pathways in CT-2A and astrocytes**. a) Influence of energy stress on mTOR activity. Cells were treated with 50 mM 2-DG for 0–30 minutes in serum free media. Cell lysates were probed with phosphorylated antibodies for mTOR and its substrate S6 kinase. b) Dephosphorylation of mTOR and S6 kinase in astrocytes was evident under energy stress condition whereas uncontrolled mTOR activity and delayed S6 kinase dephosphorylation was observed in CT-2A cells. c) Influence of rapamycin on low – glucose induced cell-death. CT-2A cells were cultured in 3.0 mM glucose containing medium with or without 50 nM rapamycin for 24 hours. Pictures were taken at 24 hours in cell culture. Note that the rapamycin treated CT-2A cells had much less floating cells than non-treated cells. Both floating and attaching cells were harvested and lysed for western blot assay for caspase – 3. Caspase-3 cleavage was noticed in CT-2A cells in low glucose condition which was prevented by rapamycin (d).

Protein synthesis is regulated by multiple cellular conditions including cellular energy levels. To determine the functional significance of AMPK phosphorylation in CT-2A tumor cells and astrocytes, we examined the mTOR/S6K protein synthesis pathway. The non-metabolizable glucose analog 2-DG is a potent glycolytic inhibitor that mimics the effects of energy starvation. We found that 2-DG (50 mM) caused a rapid dephosphorylation of mTOR and S6K in astrocytes and we also show that the dephosphorylation of S6K was delayed in CT-2A cells (Figure 4a and 4b). Furthermore, rapamycin treatment suppressed glucose deprivation-induced cell-death in the CT-2A cells (Figure 4c and 4d). Western blot analysis for apoptosis markers revealed that caspase-3 was cleaved during glucose deprivation in CT-2A and rapamycin treatment suppressed caspase-3 cleavage in CT-2A cells suggesting that sustained mTOR pathway activity is responsible for CT-2A cell-death under conditions of energy starvation (Figure 4d). These data, viewed together, indicate that the energy sensing capability of the mTOR protein synthesis pathway is less efficient in CT-2A cells than in normal astrocytes.

### Role of energy stress on LKB1 expression in normal brain

**Figure 5:**
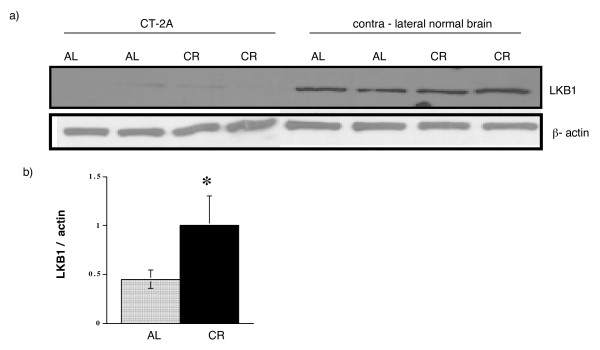
**LKB1 expression in CT-2A tumor and in contra-lateral normal brain and influence of 40%CR**. a) Influence of 40% dietary restriction on LKB1 protein expression. CT-2A tumor implantation and diet treatment was the same as mentioned above. Endogenous amount of LKB1 was analyzed. CT-2A tumor has no expression for LKB1 whereas normal brain expresses it. Moreover, 40% CR increased the expression of LKB1 in normal brain tissue. b) Ratio of LKB1 to β-actin. A significant (p < 0.05) increase in LKB1 expression has been noticed in the brain of CR mice. Tissues from 3 independent mice were analyzed.

LKB1 is the major AMPKK that responds to changes in AMP/ATP ratios. Loss of LKB1 in tumors can increase cell growth under normal energy conditions, but can enhance apoptosis under severe energy stress. On the other hand, LKB1 protects normal cells from apoptosis in response to elevated intracellular AMP. We observed that LKB1 protein was expressed in normal brain tissue, but was not expressed in the CT-2A tumor (Figure 5a). Moreover, CR significantly increased LKB1 expression in the brain (Figure 5b). Since CT-2A tumor has no functional LKB1, it is possible that another AMPKK be responsible for AMPK activation under energy stress.

### Pharmacological stimulation and inhibition of AMPK in astrocytes and CT-2A cells

**Figure 6:**
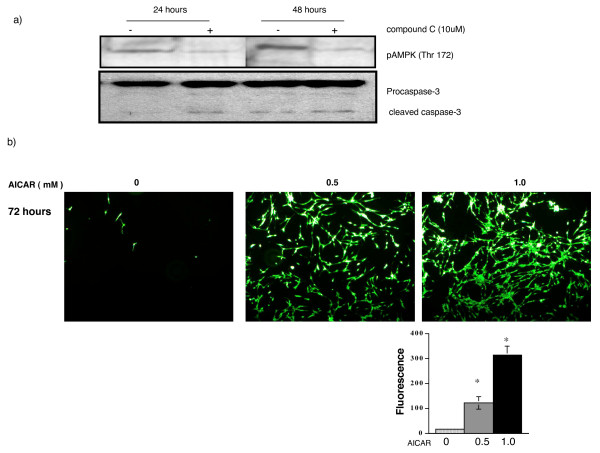
**Effects of AMPK phosphorylation in astrocytes**. a) Astrocytes were cultured with 3.0 mM glucose containing media in the presence or absence of 10 μM of compound C for 24 and 48 hours. Treatment with compound C inhibited AMPK phosphorylation and increased caspase-3 cleavage in astrocytes. b) Astrocytes were cultured with 1.0 mM glucose containing media in the presence or absence of 0.5 and 1.0 mM of AICAR for 72 hours. Treatment with AICAR significantly increased the number of astrocytes in a dose dependent manner as detected by fluorescence from calcein labeled astrocytes at p < 0.001.

To determine the role of AMPK in cultured cells we treated both CT-2A and astrocyes with AICAR and compound C in the low glucose condition. We found that astrocytes treated with 10 μM of compound C inhibited AMPK phosphorylation and increased caspase-3 cleavage after 48 hours (Figure 6a). However, compound C treated CT-2A cells had no protection from cell-death through AMPK inhibition (data not shown). Since AMPK is a critical mediator of glycolysis, it is likely that compound C induced AMPK inhibition further reduced glycolysis in CT-2A cells under low glucose condition. Therefore we didn't find any protection in compound C treated CT-2A cells from apoptosis. On the other hand, astrocytes suffered from both reduced glycolysis and inhibition of energy conserve signaling from AMPK and resulted in cell-death. Pharmacological stimulation of astrocytes with AICAR significantly increased the number of live cells under severe energy stress (Figure 6b) as detected by calcein staining and fluorescent measurement. Similar treatment to CT-2A cells, the protection was very transient under severe energy stress (data not shown). Due to reduced protein synthesis and cellular proliferation, AICAR treated astrocytes were better protected than CT-2A cells under severe energy stress condition. These data strongly suggest that normal astrocytes, due to their functional cell signaling machinery, are well protected from cell-death under energy stress but not CT-2A cells.

### TSC2 expression in CT-2A and astrocytes *in vitro *and *in vivo*: TSC2 is required for the protection of astrocytes from apoptosis under energy stress

**Figure 7:**
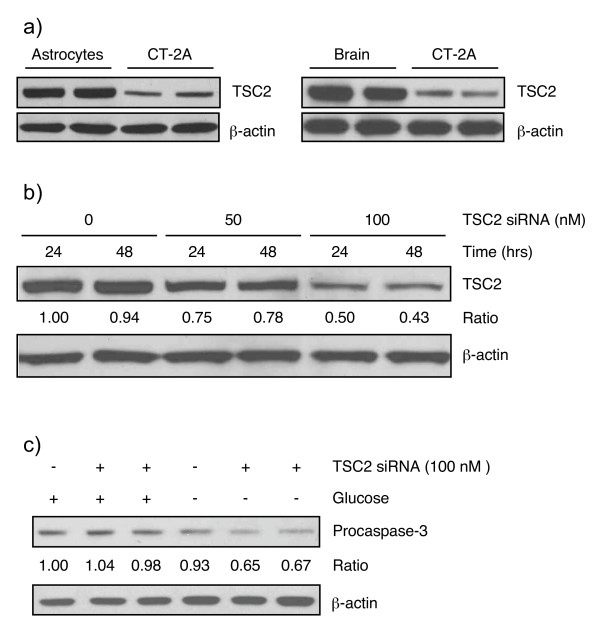
**Expression of TSC2 protein in CT-2A and astrocytes *in vitro *and *in vivo*: *Knockdown *of TSC2 reduces procaspase-3 expression in astrocytes under energy stress**. a) Western blot analysis of TSC2 protein expression. Two representative samples are shown for each cell and tissue type. CT-2A cells are deficient in TSC2 protein compared to control astrocyte or normal brain. b&c) Astrocytes were transfected with siRNA against the mouse TSC2 and starved for another 24 hours. Whole cell lysates were analyzed for procaspase-3 expression. Procaspase-3 was significantly decreased indicating increased caspase-3 cleavage and apoptosis in TSC2 deficient astrocytes compared to control cells under energy stress.

Western blot analysis was performed to examine differences in TSC2 protein expression between CT-2A cells and syngeneic mouse astrocytes as well as between tumor tissue and contra-lateral normal brain tissue from tumor-bearing mice. Figure 7a clearly shows that CT-2A tumor cells are deficient in the TSC2 protein compared with both astrocytes and normal brain tissue. To directly address the role of TSC2, as a downstream effector of AMPK, on the protection of astrocytes from energy stress induced apoptosis we treated astrocytes with mouse-specific siRNA that specifically knocked down the TSC2 gene. Incubation of astrocytes with 50 and 100 nM of TSC2 siRNA for 24 and 48 hours decreased protein expression by 25 and 50% of control non-transfected cells, respectively (Figure 7b). The 100 nM concentration was used for the energy stress experiment. The cells were first treated with 100 nM TSC2 siRNA for 36 hours and the cells were then incubated in glucose free medium for additional 24 hours. Figure 7c shows that procaspase-3 expression was reduced by 8–9% in control astrocytes that were cultured under low glucose conditions, indicating that the siRNA transfection agent could induce some death in these cells. Knockdown of TSC2 expression caused about a 40% reduction in procaspase-3 levels compared to control cells, indicating that TSC2 knockdown enhanced caspase-3 cleavage and increased apoptosis under conditions of energy stress. Overall, data presented in figure 7 suggest that TSC2 deficient astrocytes are more sensitive to death than control astrocytes under low glucose conditions.

## Discussion

Our results indicate that the response of AMPK to energy stress in brain tumors is fundamentally different from the response to a similar energy stress in the neural parenchyma. Because CT-2A cells are more highly glycolytic than normal astrocytes, the rate of ATP depletion and level of AMPK activation was greater in CT-2A cells than in normal astrocytes. Although the pro-apoptotic and the anti-apoptotic effects of AMPK are well documented, our current findings provide novel evidence that activation of AMPK produces apoptosis in brain tumor tissue and tumor cells, but prevents apoptosis in normal brain tissue and astrocytes. Our findings also suggest that LKB1, an up-stream activator of AMPK, may play a role in the anti-apoptotic effects of AMPK in normal brain under energy stress.

The coordination of cellular energy status and survival is currently a major field of interest in cell biology. Although both normal brain tissue and brain tumors are highly dependent on glucose for survival, brain tumors are more susceptible to the effects of glucose deprivation than normal brain tissue [32]. AMPK monitors the energy status of a cell and can initiate appropriate responses to ATP depletion during energy metabolic stress [2,33]. Our current findings indicate that AMPK phosphorylation is significantly greater in CT-2A astrocytoma cells than in normal astrocytes after 24 hr of glucose deprivation. The enhanced AMPK phosphorylation is associated with death in the CT-2A cells, but not in the astrocytes under normal energy condition. These findings are also consistent with our *in vivo *observations where a 40% dietary restriction, which lowers blood glucose levels, caused a significant elevation of AMPK phosphorylation in both tumor and in the brain. AMPK phosphorylation was associated with caspase 3 cleavage in CT-2A brain tumor, but not in contra-lateral normal appearing brain tissue.

Previous studies showed that AMPK activation with either AICAR or low glucose causes apoptosis in cultured rat b cells or in insulin producing MIN6 cells [13,14]. The pro-apoptotic effects of AMPK activation in various cancer cells includes inhibition of fatty acid synthase and induction of stress kinase and caspase-3 [7-9,14,17]. On the other hand, AMPK activation also had a protective effect on stress injured cells in heart ischemia and reperfusion injury [34,11,35]. In contrast to CT-2A cells under energy stress where AMPK activation was associated with enhanced apoptosis, we found that AMPK activation protected normal astrocytes form apoptosis under energy stress. This came from findings that compound C, an inhibitor of AMPK activation, enhanced astrocyte apoptosis under energy stress. On the other hand, stimulation of astrocytes with AMPK stimulator significantly increased the number of astrocytes under very low glucose conditions. A linkage between the LKB1 and the TSC2 tumor suppressors may account in part for the apparent opposite effects of AMPK activation on cell survival and cell-death.

LKB1 is an upstream AMPKK which phosphorylates and activates AMPK under normal physiological conditions [6,36]. Mutations of LKB1 occur in Peutz-Jeghers syndrome and enhance susceptibility to tumor formation [18-20]. Shaw et al [6] found that LKB1 deficient cells are hypersensitive to apoptosis induced by energy stress. On the other hand, it is essential to protect cells from apoptosis in response to agents that elevate intracellular AMP. The loss of LKB1 in tumors can result in increased cell growth, but cells lacking LKB1 are resistant to transformation and readily undergo apoptosis under energy stress condition [6]. We found that LKB1 is not expressed in CT-2A tumor tissue, but is expressed in normal brain tissue contra-lateral to the tumor. We also found that 40% dietary restriction caused a significant elevation of LKB1 expression in normal brain tissue. The lack of LKB1 in CT-2A tumor cells might explain the aberrant growth of these cells under *in vivo *and *in vitro *conditions. Since LKB1 is absent in CT-2A tumor, other AMPKKs could be responsible for the stimulation of AMPK under energy stress. Hurley et al [24] investigated other AMPKK in three LKB1 deficient cancer cell lines and found that Ca2+/calmodulin dependent protein kinase kinases regulated AMPK activity in those cell lines. Rattan et al [37] found that AMPK can be a potential target for treatment of various cancers independent of the functional tumor suppressor genes, LKB1. In our findings the presence of LKB1 in normal brain and induction of apoptosis by inhibiting AMPK in normal astrocyte potentially suggest that LKB1/AMPK may play a role in their survival under energy stress.

Cell survival under energy stress is dependent in part on the ability to conserve energy through inhibition of protein synthesis. Protein synthesis utilizes approximately 20%–25% of the total cellular energy and is coordinated with cellular energy status [38]. Activation of AMPK inhibits protein synthesis by suppressing the functions of multiple translation regulators including S6K, 4E-BP1, and eEF2 in response to energy starvation [26,39]. The mechanism of S6K inhibition by AMPK involves the mammalian target of rapamycin (mTOR) pathway [27,40-43]. We found that 2-DG inhibited both mTOR and S6K phosphorylation in normal astrocytes, but not in CT-2A cells. Furthermore, rapamycin treatment suppressed glucose deprivation induced cell-death in CT-2A cells suggesting that high mTOR activity is responsible for cell-death under energy starvation condition. Our results support the previous findings of Inoki et al [26] who found that rapamycin treatment suppressed glucose-deprivation induced cell-death in TSC2 -/- epithelial cells.

Like LKB1, TSC2 is another tumor suppressor gene that influences protein synthesis downstream of AMPkinase and upstream of mTOR [26,44,45]. Inoki et al [26] proposed that phosphorylation of TSC2 by AMPK protect cells from energy deprivation induced cell-death. In our study, TSC2 *knockdown *astrocytes are hypersensitive to apoptosis under energy stress condition. Absence of both LKB1 and TSC2 in CT-2A cells well explain the uncontrolled mTOR activity and lack of protection from cell-death under severe energy stress. Shaw et al [27] also found that LKB1 is required for repression of mTOR under low ATP conditions in cultured cells in an AMPK and TSC2 dependent manner and LKB1 mutant mice show elevated signaling downstream of mTOR. These observations together with our findings suggest that the LKB1-AMPK-TSC2-mTOR pathway underlies the adaptive ability of normal cells to conserve energy when food is scarce.

**Figure 8:**
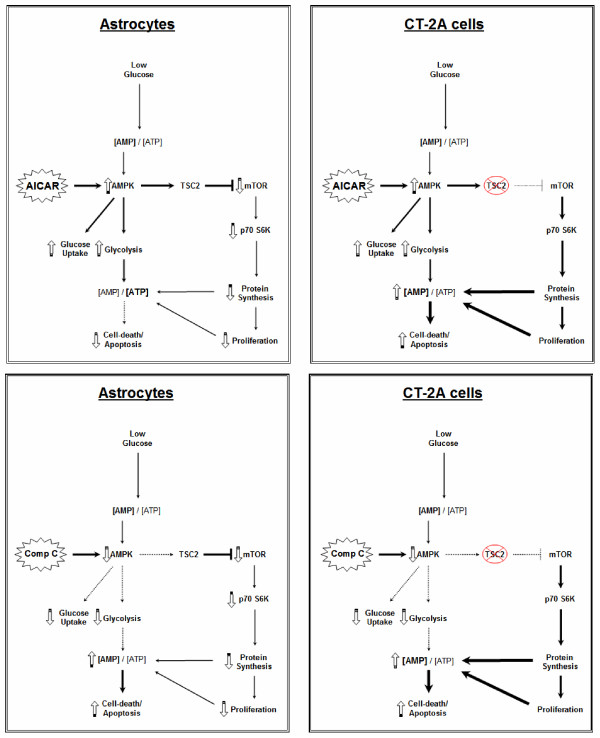
**Possible role of AICAR and compound C on the AMPK pathway and on glycolysis in CT-2A and astrocytes under low glucose conditions**. AICAR stimulates AMPK phosphorylation and glycolysis by increasing glucose uptake in the cells. Under low glucose conditions, increased AMP/ATP ratio stimulates AMPK and glycolysis both in astrocytes and CT-2A. The presence of an intact TSC2- mTOR signaling pathway in astrocytes helps to reduce protein sysnthesis to conserve energy and thus prevents apoptosis. Sustained mTOR-S6 kinase pathway activity enhances apoptosis in TSC2 deficient CT-2A cells under low glucose conditions. compound C, on the other hand, inhibits AMPK phosphorylation and glycolysis in the cells. Thus, although normal astrocytes downregulate protein synthesis under low glucose conditions as an adaptation to conserve energy for survival, the decrease in glycolytic ATP production due to compound C results in increased apoptosis.

AMPK has been shown to be a key mediator of glycolysis [46,47]. Pharmacological stimulation of AMPK with the AMP analogue AICAR enhances glucose uptake and glycolysis, and inhibition of AMPK with compound C, on the other hand, reduces glycolysis [48]. This phenomenon is exemplified in cells deficient in the TSC2 protein. We show in the current study that the treatment of the TSC2-deficient CT-2A cells with AICAR increases apoptosis and is associated with enhanced glycolysis and sustained elevated levels of proliferation when the cells are cultured under normal glucose (figure 2) or low glucose conditions (data not shown). Under low glucose conditions, treatment of CT-2A cells with compound C further reduced glycolysis and therefore we didn't find any rescue from apoptosis (data not shown). Similarly, normal mouse astrocytes are not protected from death when cultured under similar conditions, suggesting that the presence of an intact AMPK-TSC2-mTOR pathway is necessary for cells to conserve energy for survival, especially when glycolysis is inhibited (figure 8).

We previously showed that moderate CR (40%) enhanced apoptosis in experimental mouse and human brain tumors while enhancing the overall health and vitality of the tumor-bearing mice [49]. Though the pro-apoptotic effects of CR in brain tumors occur in large part from reduced glycolytic energy that tumors rely upon for growth, its effects on normal brain were not clear. Also, the molecular mechanisms involved in the global therapeutic approach of CR in brain tumors were unknown. We also proposed that alternative energy fuels like ketones for the protection of normal brain under energy stress condition [32]. The bioenergetic transition from glucose to ketones during CR might have a direct influence on AMPK activation and cell survival for the normal brain.

## Conclusion

In conclusion, we provide evidence for a dual role of AMPK activation in the protection of normal brain from energy stress while also killing the brain tumor cells. Further studies are required to better define the role of AMPK as a potential regulator of energy metabolism and apoptosis in normal and tumor tissues.

## Methods

### Reagents

Phosphorylated and non – phosphorylated AMPK(Thr172), mTOR(Ser2448), pS6 kinase (Thr389), caspase-9, caspase-3, PARP, cytochrome-c antibodies, lysis buffer (10×), and rapamycin (mTOR inhibitor) were purchased from Cell Signaling, MA. Anti LKB1 was purchased from SantaCruz Biotechnology, CA. β-actin was purchased from Novus Biologicals, CO. 2 deoxyglucose (2-DG) from Sigma, MO, AICAR (AMPK stimulator), compound C (AMPK inhibitor) was from Calbiochem, CA. Calcein was purchased from Molecular Probe (Carlsbad, CA).

### Cell culture conditions

The CT-2A, mouse astrocytoma cell line was established as previously described [50]. The C8-D1A, astrocyte cell line was purchased from American Type Culture Collection (ATCC), VA. Both CT-2A tumor and astrocyte cell lines were originated from C57BL/6J mice. Cell lines were maintained in Dulbecco's modified Eagle's medium (DMEM) supplemented with 10% fetal bovine serum and 0.5% penicillin/streptomycin (Sigma, St. Louis, MO). The cells were cultured in a CO_2 _incubator with a humidified atmosphere containing 95% air and 5% CO_2 _at 37°C. For ATP depletion, the cells were stimulated with 2-DG and different amount of glucose and glutamine containing media. For 2-DG treatment, the culture media was replaced with serum free DMEM containing 6 mM glucose 24 hours before 2-DG stimulation. The cells were then stimulated with 50 mM of 2-DG that was dissolved in serum free DMEM for indicated times. For glucose starvation, the cells were pretreated with serum free DMEM for 24 hours and were then cultured with 6.0 mM glucose and/or 4.0 mM glutamine and, 0 mM glucose and 0 mM glutamine in serum free DMEM for 24 hours. Additionally, 3.0 mM glucose and 2.0 mM glutamine, 1.0 mM glucose and 0.66 mM of glutamine in serum free DMEM were used for indicated time.

For rapamycin treatment, CT-2A cells were cultured with serum free media for 24 hours and were then incubated with 3.0 mM glucose containing media in the presence or absence of 20 nM rapamycin for 24 hours. For compound C treatment, astrocytes were cultured in 3.0 mM glucose containing media in the presence or absence of 1.0 μM compound C for 24 and 48 hours. For AICAR treatment, CT-2A and astrocytes were cultured in 6.0 mM glucose containing media for 24 hours and in 1.0 mM glucose containing for 72 hours in the presence and absence of 0.5 and 1.0 mM AICAR.

To harvest cells, the flasks were washed once with phosphate buffered saline (PBS) and treated with trypsin-EDTA for 2 minutes. Cells were gently pipetted off the flask with PBS, transferred to 15 ml conical centrifuge tubes, centrifuged for 3 minutes at 1000 rpm, and pellets were washed again with PBS. Final pelletes were lysed with lysis buffer and were centrifuged at 8,100 × g for 20 min at 4°C. The lysates were collected and stored at -80°C for protein analysis. For caspase analysis, both the floating and the adhered cells were collected and lysed. ATP determination – Five thousand cells per well were seeded in 96 wells plate and allowed to adhere for 24 hours. Cells were washed with serum free media and replaced with fresh media with 6.0 mM glucose and without glucose. ATP levels were measured using the ViaLight Plus kit (Cambrex, Charlescity, IA) that is based on the bioluminescent measurement of ATP in presence of luciferase, Mg^2+ ^and O_2_. Luminescence was measured with a microplate luminometer with an integration time of 5s/well.

### siRNA transfection

siRNA against the mouse TSC2 (mouse) was bought from SantaCruz and they were transfected using Lipofectamine 2000 (Invitrogen, CA). Transfections were carried out using the concentrations of 50 and 100 nM for 24 and 48 hours. For each experiment astrocytes were grown for 36–48 hours with siRNA, transfected and control astrocytes were incubated for additional 24 hours in the presence and absence of glucose and glutamine and cell lysates were collected for western blot.

### Western Blot Analysis

Tumor cells and tissues were homogenized in ice-cold lysis buffer (Cell Signaling Technology, MA) containing 20 mM Tris-HCl (pH 7.5), 150 mM NaCl, 1 mM Na_2_EDTA, 1 mM EGTA, 1% Triton, 2.5 mM NaPP_i_, 1 mM α-glycerophosphate, 1 mM Na_3 _VO_4_, 1 μg/ml leupeptin, and 1 mM phenylmethylsufonyl fluoride. Tissue lysates were transferred to Eppendorf tubes, mixed on a rocker for 1 hr at 4°C, and then centrifuged at 8,100 × g for 20 min. Supernatants were collected and protein concentrations were estimated using the Bio-Rad DC protein assay. Approximately 30–50 μg of total protein from cells and 10–20 μg of protein from tissues for each sample were loaded on a 12% SDS-polyacrylamide gel (Bio-Rad, CA) and electrophoresed. Proteins were transferred to a PVDF immobilon TM-P membrane (Millipore, MA) overnight at 4°C and blocked in 5% nonfat powered milk in Tris-buffered saline with Tween 20 (pH 7.6) for 3 h. Blots were then probed with different indicated antibodies overnight at 4°C with gentle shaking. The blots were then incubated with appropriate whole horseradish peroxidase-conjugated secondary antibody at room temperature. Bands were visualized using enhanced chemiluminescence plus system (Amersham, UK). Blots were reprobed with β-actin antibody used as a loading control.

### Measurement of apoptosis by Annexin V staining

CT-2A and astrocyte cells (including floating cells) grown in 6 well plates were collected following mild trypsinization. According to manufacturer's (BD Bioscience, Pharmingen, CA) protocol, trypsinized cells were washed once with PBS, and they were resuspended in 100 ul of Annexin binding buffer mixed with 5 ul of Annexin V fluorescein conjugate and propidium Iodide (PI). Resuspended cells were incubated at room temperature in the dark for 15 minutes. Labeled cells were analyzed by FACS (Beckman coulter, CA).

### Mice and experimental brain tumors

Mice of the C57BL/6J (B6) strain were obtained from the Jackson Laboratory (Bar Harbor, ME). The mice were propagated in the animal care facility of the Department of Biology of Boston College, using the animal husbandry conditions described previously [51]. The syngeneic mouse brain tumor, CT-2A, was originally produced by implantation of a chemical carcinogen, 20-methylcholanthrene, into the brains of B6 mice [50,52]. The CT-2A tumor arose in the cerebral cortex and was characterized as a malignant anaplastic astrocytoma [50]. The morphological, biochemical, and growth characteristics of the CT-2A brain tumor have been previously described [50]. Male B6 mice (8–12 weeks of age) were used as tumor recipients. All animal experiments were carried out with the ethical committee approval in accordance with the National Institutes of Health Guide for the Care and Use of Laboratory Animals and approved by the Institutional Care Committee.

### Brain tumor implantation

Briefly, B6 mice were anaesthetized with Avertin (0.1 ml/10 g body weight) intra-peritoneally and their heads shaved and swabbed with 70% ethyl alcohol under sterile conditions. Small tumor pieces (about 1 mm^3^, estimated using a 1 mm × 1 mm grid) from the donor tumor were implanted into the right cerebral hemisphere of anaesthetized recipient mice as we previously described [49,53].

### Caloric restriction

The mice were group housed prior to the initiation of the experiment and were then separated and randomly assigned to either a control group that was fed *ad libitum *(AL) or to a caloric restricted (CR) group that was fed a total CR of 40% (60% of the control group). Within each experiment, the AL-fed and CR-fed mice were matched for age and body weight. Each mouse was housed singly in a plastic shoebox cage with a filter top and was given a cotton nesting-pad for warmth. CR was initiated 24 hours after tumor implantation and was continued for 13 days after implantation. Total CR maintains a constant ratio of nutrients to energy, i.e., the average daily food intake (grams) for the AL fed mice was determined every other day and the CR-fed mice were given 60% of that quantity on a daily basis [54,55]. All mice received PROLAB RMH 3000 chow (LabDiet, Purina, Richmond, IN) that contained a balance of mouse nutritional ingredients and delivered 4.1–4.4 Kcal/g gross energy according to the manufacturer's specifications. Body weights of all the mice were recorded every other day.

### *In situ *apoptotic cell detection (TUNEL)

Apoptotic cells were detected using the ApopTag *in situ *detection kit TUNEL (terminal deoxynucleotidyl transferase mediated deoxyuridine triphosphate biotin nick end labeling) (Oncor, Gaithersberg, MD) as we previously described [49,55].

## List of abbreviations

AMPK: AMP-activated protein kinase; AMPKK: AMP-activated protein kinase kinase; mTOR: mammalian target of rapamycin; 2-DG: 2-deoxyglucose; DMEM: Dulbecco's modified Eagle's medium; CR: caloric restriction; TUNEL: terminal deoxynucleotidyl transferase-mediated dUTP nick end labeling.

## Competing interests

The authors declare that they have no competing interests.

## Authors' contributions

PM designed and performed the research, analyzed the data, and wrote the paper; TJM performed the research; JM performed the research, analyzed the data, and helped in drafting the manuscript; DB helped in the collection of flow cytometry data; TCC provided critical comments for the study; TNS provided supports for the work and also provided critical comments in the drafting of the manuscript. All authors approved the final version of the manuscript.

## References

[B1] Hardie DG, Scott JW, Pan DA, Hudson ER (2003). Management of cellular energy by the AMP-activated protein kinase system. FEBS Lett.

[B2] Kemp BE, Stapleton D, Campbell DJ, Chen ZP, Murthy S, Walter M, Gupta A, Adams JJ, Katsis F, van Denderen B, Jennings IG, Iseli T, Michell BJ, Witters LA (2003). AMP-activated protein kinase, super metabolic regulator. Biochem Soc Trans.

[B3] Hawley SA, Davison M, Woods A, Davies SP, Beri RK, Carling D, Hardie DG (1996). Characterization of the AMP-activated protein kinase kinase from rat liver and identification of threonine 172 as the major site at which it phosphorylates AMP-activated protein kinase. J Biol Chem.

[B4] Hamilton SR, O'Donnell JB, Hammet A, Stapleton D, Habinowski SA, Means AR, Kemp BE, Witters LA (2002). AMP-activated protein kinase kinase: detection with recombinant AMPK alpha1 subunit. Biochem Biophys Res Commun.

[B5] Woods A, Vertommen D, Neumann D, Turk R, Bayliss J, Schlattner U, Wallimann T, Carling D, Rider MH (2003). Identification of phosphorylation sites in AMP-activated protein kinase (AMPK) for upstream AMPK kinases and study of their roles by site-directed mutagenesis. J Biol Chem.

[B6] Shaw RJ, Kosmatka M, Bardeesy N, Hurley RL, Witters LA, DePinho RA, Cantley LC (2004). The tumor suppressor LKB1 kinase directly activates AMP-activated kinase and regulates apoptosis in response to energy stress. Proc Natl Acad Sci USA.

[B7] Saitoh M, Nagai K, Nakagawa K, Yamamura T, Yamamoto S, Nishizaki T (2004). Adenosine induces apoptosis in the human gastric cancer cells via an intrinsic pathway relevant to activation of AMP-activated protein kinase. Biochem Pharmacol.

[B8] Li J, Jiang P, Robinson M, Lawrence TS, Sun Y (2003). AMPK-beta1 subunit is a p53-independent stress responsive protein that inhibits tumor cell growth upon forced expression. Carcinogenesis.

[B9] Xiang X, Saha AK, Wen R, Ruderman NB, Luo Z (2004). AMP-activated protein kinase activators can inhibit the growth of prostate cancer cells by multiple mechanisms. Biochem Biophys Res Commun.

[B10] Blazquez C, Geelen MJ, Velasco G, Guzman M (2001). The AMP-activated protein kinase prevents ceramide synthesis de novo and apoptosis in astrocytes. FEBS Lett.

[B11] Stefanelli C, Stanic I, Bonavita F, Flamigni F, Pignatti C, Guarnieri C, Caldarera CM (1998). Inhibition of glucocorticoid-induced apoptosis with 5-aminoimidazole-4-carboxamide ribonucleoside, a cell-permeable activator of AMP-activated protein kinase. Biochem Biophys Res Commun.

[B12] Durante P, Gueuning MA, Darville MI, Hue L, Rousseau GG (1999). Apoptosis induced by growth factor withdrawal in fibroblasts overproducing fructose 2,6-bisphosphate. FEBS Lett.

[B13] Kefas BA, Heimberg H, Vaulont S, Meisse D, Hue L, Pipeleers D, Casteele M Van de (2003). AICA-riboside induces apoptosis of pancreatic beta cells through stimulation of AMP-activated protein kinase. Diabetologia.

[B14] Kefas BA, Cai Y, Ling Z, Heimberg H, Hue L, Pipeleers D, Casteele M Van de (2003). AMP-activated protein kinase can induce apoptosis of insulin-producing MIN6 cells through stimulation of c-Jun-N-terminal kinase. J Mol Endocrinol.

[B15] Dagon Y, Avraham Y, Berry EM (2006). AMPK activation regulates apoptosis, adipogenesis, and lipolysis by eIF2alpha in adipocytes. Biochem Biophys Res Commun.

[B16] Dagon Y, Avraham Y, Magen I, Gertler A, Ben-Hur T, Berry EM (2005). Nutritional status, cognition, and survival: a new role for leptin and AMP kinase. J Biol Chem.

[B17] Meisse D, Casteele M Van de, Beauloye C, Hainault I, Kefas BA, Rider MH, Foufelle F, Hue L (2002). Sustained activation of AMP-activated protein kinase induces c-Jun N-terminal kinase activation and apoptosis in liver cells. FEBS Lett.

[B18] Boudeau J, Sapkota G, Alessi DR (2003). LKB1, a protein kinase regulating cell proliferation and polarity. FEBS Lett.

[B19] Bardeesy N, Sinha M, Hezel AF, Signoretti S, Hathaway NA, Sharpless NE, Loda M, Carrasco DR, DePinho RA (2002). Loss of the Lkb1 tumour suppressor provokes intestinal polyposis but resistance to transformation. Nature.

[B20] Miyoshi H, Nakau M, Ishikawa TO, Seldin MF, Oshima M, Taketo MM (2002). Gastrointestinal hamartomatous polyposis in Lkb1 heterozygous knockout mice. Cancer Res.

[B21] Moore P (2003). Connecting LKB1 and AMPK links metabolism with cancer. Journal of Biology.

[B22] Hawley SA, Boudeau J, Reid JL, Mustard KJ, Udd L, Makela TP, Alessi DR, Hardie DG (2003). Complexes between the LKB1 tumor suppressor, STRAD alpha/beta and MO25 alpha/beta are upstream kinases in the AMP-activated protein kinase cascade. J Biol.

[B23] Williamson B, Coniglio JG (1971). The effects of pyridoxine deficiency and of caloric restriction on lipids in the developing rat brain. J Neurochem.

[B24] Hurley RL, Anderson KA, Franzone JM, Kemp BE, Means AR, Witters LA (2005). The Ca2+/calmodulin-dependent protein kinase kinases are AMP-activated protein kinase kinases. J Biol Chem.

[B25] Woods A, Dickerson K, Heath R, Hong SP, Momcilovic M, Johnstone SR, Carlson M, Carling D (2005). Ca2+/calmodulin-dependent protein kinase kinase-beta acts upstream of AMP-activated protein kinase in mammalian cells. Cell Metab.

[B26] Inoki K, Zhu T, Guan KL (2003). TSC2 mediates cellular energy response to control cell growth and survival. Cell.

[B27] Shaw RJ, Bardeesy N, Manning BD, Lopez L, Kosmatka M, DePinho RA, Cantley LC (2004). The LKB1 tumor suppressor negatively regulates mTOR signaling. Cancer Cell.

[B28] Kwiatkowski DJ, Zhang H, Bandura JL, Heiberger KM, Glogauer M, el-Hashemite N, Onda H (2002). A mouse model of TSC1 reveals sex-dependent lethality from liver hemangiomas, and up-regulation of p70S6 kinase activity in Tsc1 null cells. Hum Mol Genet.

[B29] Ramaswamy S, Nakamura N, Vazquez F, Batt DB, Perera S, Roberts TM, Sellers WR (1999). Regulation of G1 progression by the PTEN tumor suppressor protein is linked to inhibition of the phosphatidylinositol 3-kinase/Akt pathway. Proc Natl Acad Sci USA.

[B30] Neshat MS, Mellinghoff IK, Tran C, Stiles B, Thomas G, Petersen R, Frost P, Gibbons JJ, Wu H, Sawyers CL (2001). Enhanced sensitivity of PTEN-deficient tumors to inhibition of FRAP/mTOR. Proc Natl Acad Sci USA.

[B31] Podsypanina K, Lee RT, Politis C, Hennessy I, Crane A, Puc J, Neshat M, Wang H, Yang L, Gibbons J, Frost P, Dreisbach V, Blenis J, Gaciong Z, Fisher P, Sawyers C, Hedrick-Ellenson L, Parsons R (2001). An inhibitor of mTOR reduces neoplasia and normalizes p70/S6 kinase activity in Pten+/- mice. Proc Natl Acad Sci USA.

[B32] Seyfried TN, Mukherjee P (2005). Targeting energy metabolism in brain cancer: review and hypothesis. Nutr Metab (Lond).

[B33] Davies SP, Carling D, Hardie DG (1989). Tissue distribution of the AMP-activated protein kinase, and lack of activation by cyclic-AMP-dependent protein kinase, studied using a specific and sensitive peptide assay. Eur J Biochem.

[B34] Russell RR, Li J, Coven DL, Pypaert M, Zechner C, Palmeri M, Giordano FJ, Mu J, Birnbaum MJ, Young LH (2004). AMP-activated protein kinase mediates ischemic glucose uptake and prevents postischemic cardiac dysfunction, apoptosis, and injury. J Clin Invest.

[B35] Nishino Y, Miura T, Miki T, Sakamoto J, Nakamura Y, Ikeda Y, Kobayashi H, Shimamoto K (2004). Ischemic preconditioning activates AMPK in a PKC-dependent manner and induces GLUT4 up-regulation in the late phase of cardioprotection. Cardiovasc Res.

[B36] Woods A, Johnstone SR, Dickerson K, Leiper FC, Fryer LG, Neumann D, Schlattner U, Wallimann T, Carlson M, Carling D (2003). LKB1 is the upstream kinase in the AMP-activated protein kinase cascade. Curr Biol.

[B37] Rattan R, Giri S, Singh AK, Singh I (2005). 5-Aminoimidazole-4-carboxamide-1-beta-D-ribofuranoside inhibits cancer cell proliferation in vitro and in vivo via AMP-activated protein kinase. J Biol Chem.

[B38] Schmidt EV (1999). The role of c-myc in cellular growth control. Oncogene.

[B39] Horman S, Browne G, Krause U, Patel J, Vertommen D, Bertrand L, Lavoinne A, Hue L, Proud C, Rider M (2002). Activation of AMP-activated protein kinase leads to the phosphorylation of elongation factor 2 and an inhibition of protein synthesis. Curr Biol.

[B40] Kimura N, Tokunaga C, Dalal S, Richardson C, Yoshino K, Hara K, Kemp BE, Witters LA, Mimura O, Yonezawa K (2003). A possible linkage between AMP-activated protein kinase (AMPK) and mammalian target of rapamycin (mTOR) signalling pathway. Genes Cells.

[B41] Krause U, Bertrand L, Hue L (2002). Control of p70 ribosomal protein S6 kinase and acetyl-CoA carboxylase by AMP-activated protein kinase and protein phosphatases in isolated hepatocytes. Eur J Biochem.

[B42] Fingar DC, Richardson CJ, Tee AR, Cheatham L, Tsou C, Blenis J (2004). mTOR controls cell cycle progression through its cell growth effectors S6K1 and 4E-BP1/eukaryotic translation initiation factor 4E. Mol Cell Biol.

[B43] Cheng SW, Fryer LG, Carling D, Shepherd PR (2004). Thr2446 is a novel mammalian target of rapamycin (mTOR) phosphorylation site regulated by nutrient status. J Biol Chem.

[B44] Inoki K, Li Y, Zhu T, Wu J, Guan KL (2002). TSC2 is phosphorylated and inhibited by Akt and suppresses mTOR signalling. Nat Cell Biol.

[B45] Goncharova EA, Goncharov DA, Eszterhas A, Hunter DS, Glassberg MK, Yeung RS, Walker CL, Noonan D, Kwiatkowski DJ, Chou MM, Panettieri RA, Krymskaya VP (2002). Tuberin regulates p70 S6 kinase activation and ribosomal protein S6 phosphorylation. A role for the TSC2 tumor suppressor gene in pulmonary lymphangioleiomyomatosis (LAM). J Biol Chem.

[B46] Almeida A, Moncada S, Bolanos JP (2004). Nitric oxide switches on glycolysis through the AMP protein kinase and 6-phosphofructo-2-kinase pathway. Nat Cell Biol.

[B47] Almeida A, Almeida J, Bolanos JP, Moncada S (2001). Different responses of astrocytes and neurons to nitric oxide: the role of glycolytically generated ATP in astrocyte protection. Proc Natl Acad Sci USA.

[B48] Wu M, Neilson A, Swift AL, Moran R, Tamagnine J, Parslow D, Armistead S, Lemire K, Orrell J, Teich J, Chomicz S, Ferrick DA (2007). Multiparameter metabolic analysis reveals a close link between attenuated mitochondrial bioenergetic function and enhanced glycolysis dependency in human tumor cells. Am J Physiol Cell Physiol.

[B49] Mukherjee P, Abate LE, Seyfried TN (2004). Antiangiogenic and proapoptotic effects of dietary restriction on experimental mouse and human brain tumors. Clin Cancer Res.

[B50] Seyfried TN, El-Abbadi M, Roy ML (1992). Ganglioside distribution in murine neural tumors. Mol Chem Neuropathol.

[B51] Flavin HJ, Wieraszko A, Seyfried TN (1991). Enhanced aspartate release from hippocampal slices of epileptic (El) mice. J Neurochem.

[B52] Zimmerman HM, Arnold H (1941). Experimental brain tumors: I. tumors produced with methylcholanthrene. Cancer Res.

[B53] Ranes MK, El-Abbadi M, Manfredi MG, Mukherjee P, Platt FM, Seyfried TN (2001). N -butyldeoxynojirimycin reduces growth and ganglioside content of experimental mouse brain tumours. Br J Cancer.

[B54] Mukherjee P, Sotnikov AV, Mangian HJ, Zhou JR, Visek WJ, Clinton SK (1999). Energy intake and prostate tumor growth, angiogenesis, and vascular endothelial growth factor expression. J Natl Cancer Inst.

[B55] Mukherjee P, El-Abbadi MM, Kasperzyk JL, Ranes MK, Seyfried TN (2002). Dietary restriction reduces angiogenesis and growth in an orthotopic mouse brain tumour model. Br J Cancer.

